# Troponin Elevation in Older Patients with Acute Pneumonia: Frequency and Prognostic Value

**DOI:** 10.3390/jcm9113623

**Published:** 2020-11-10

**Authors:** Alain Putot, Emmanuel Bouhey, Jennifer Tetu, Jérémy Barben, Eléonore Timsit, Sophie Putot, Patrick Ray, Patrick Manckoundia

**Affiliations:** 1 Geriatrics Internal Medicine Department, Centre Hospitalier Universitaire Dijon Bourgogne, 21000 Dijon, France; jeremy.barben@chu-dijon.fr (J.B.); sophie.putot@chu-dijon.fr (S.P.); patrick.manckoundia@chu-dijon.fr (P.M.); 2 Physiopathologie et Epidémiologie Cérébro-Cardiovasculaires (PEC2), EA 7460, Université Bourgogne Franche Comté, 21000 Dijon, France; 3 Emergency Department, Centre Hospitalier Universitaire Dijon Bourgogne, 21000 Dijon, France; emmanuel.bouhey@chu-dijon.fr (E.B.); eleonore.timsit@chu-dijon.fr (E.T.); patrick.ray@chu-dijon.fr (P.R.); 4 Microbiology Department, Centre Hospitalier Universitaire Dijon Bourgogne, 21000 Dijon, France; jennifer.tetu@chu-dijon.fr

**Keywords:** aged, mortality, myocardial infarction, myocardial injury, pneumonia, troponin

## Abstract

Cardiovascular (CV) events are particularly frequent after acute pneumonia (AP) in the elderly. We aimed to assess whether cardiac troponin I, a specific biomarker of myocardial injury, independently predicts CV events and death after AP in older inpatients. Among 214 consecutive patients with AP aged ≥75 years admitted to a university hospital, 171 with a cardiac troponin I sample in the 72 h following diagnosis of AP were included, and 71 (42%) were found to have myocardial injury (troponin > 100 ng/L). Patients with and without myocardial injury were similar in terms of age, gender and comorbidities, but those with myocardial injury had more severe clinical presentation (median (interquartile range) Pneumonia Severity Index: 60 (40–95) vs. 45 (30–70), *p* = 0.003). Myocardial injury was strongly associated with in-hospital myocardial infarction (25% vs. 0%, *p* < 0.001), CV mortality (11 vs. 1%, *p* = 0.003) and all-cause mortality (34 vs. 13%, *p* = 0.002). After adjustment for confounders, myocardial injury remained a strong predictive factor of in-hospital mortality (odds ratio (95% confidence interval): 3.32 (1.42–7.73), *p* = 0.005) but not one-year mortality (1.61 (0.77–3.35), *p* = 0.2). Cardiac troponin I elevation, a specific biomarker of myocardial injury, was found in nearly half of an unselected cohort of older inpatients with AP and was associated with a threefold risk of in-hospital death.

## 1. Introduction

Acute pneumonia (AP) is a major medical issue in the elderly population. It is the second leading cause of hospitalization behind heart failure [1] and is a leading cause of death. The mortality rate is estimated to be up to 30% in the very old. Age and comorbidities greatly increase the risk of death: in Europe, approximately 90% of deaths due to pneumonia occur in people aged >65 years [2]. Up to 75% of older patients with AP require hospitalization [3]. Though the choice of appropriate antibiotic therapy is a key issue in the acute phase of AP, the long-term prognosis in the elderly population mainly depends on comorbidity-related decompensation [4]. Excess risk of mortality is highest in the first week after AP but persists for several months [5], mainly as a result of cardiovascular (CV) events [6,7,8]. The prothrombotic effects of AP pathogens have been demonstrated in several experimental studies [9,10,11,12], but acute mismatch between myocardial oxygen supply and demand due to hypoxemia and sepsis also frequently leads to type 2 myocardial infarction (MI) [13]. In a meta-analysis of observational studies, CV complications were found in 14% of AP patients [14]. However, CV risk is highly influenced by age and comorbidities [15]. Distinguishing between myocardial infarction (especially type 2 myocardial infarction) and other recently redefined causes of myocardial injury [16] is often clinically challenging. However, this distinction is needed for a deeper understanding and better prediction, prevention and management of myocardial injury events [17], which are especially frequent after AP.

Among the keys to establishing preventive strategies and optimizing medical management are the stratification of mortality risk and the identification of patients most susceptible to CV complications in the short and long-term after AP.

A large proportion of AP inpatients have elevated levels of cardiac troponin, indicating that AP is often complicated by myocardial injury, a phenomenon which may be predictive of poor outcomes [18]. However, the prognostic value of troponin elevation after AP has not been fully evaluated so far [18,19,20]. Even though older patients have a particularly high risk of developing AP and post-AP CV events [21], justifying local recommendations for systematic screening in our hospital, no study has specifically evaluated the frequency and prognostic value of myocardial injury after AP in this population. In this unselected cohort of patients aged ≥75 years hospitalized for AP, we hypothesized that myocardial injury would be an independent factor of worse prognosis in the short and long term.

## 2. Methods

### 2.1. Study Design and Population

The clinical records of all patients aged 75 and older who were admitted for AP through the emergency department of the Dijon University Hospital between 1 January and 30 June 2013, were retrospectively reviewed. Eligible participants were identified through the diagnostic coding system in the French medical information database. In total, a primary or secondary diagnosis of AP was identified and retained by the treating physician in 279 patients. The records were reviewed for eligibility by a study coordinator. A diagnosis of AP was retained according to the following criteria, as defined by the current American guidelines [22]: (1) two or more of the following signs: new cough, sputum production, dyspnea, pleuritic pain, abnormal temperature (<35.6 °C or >37.8 °C), altered breathing sounds on auscultation; and (2) a new infiltrate on chest imaging. Patients with ventilator-associated pneumonia were not included.

**Figure 1:**
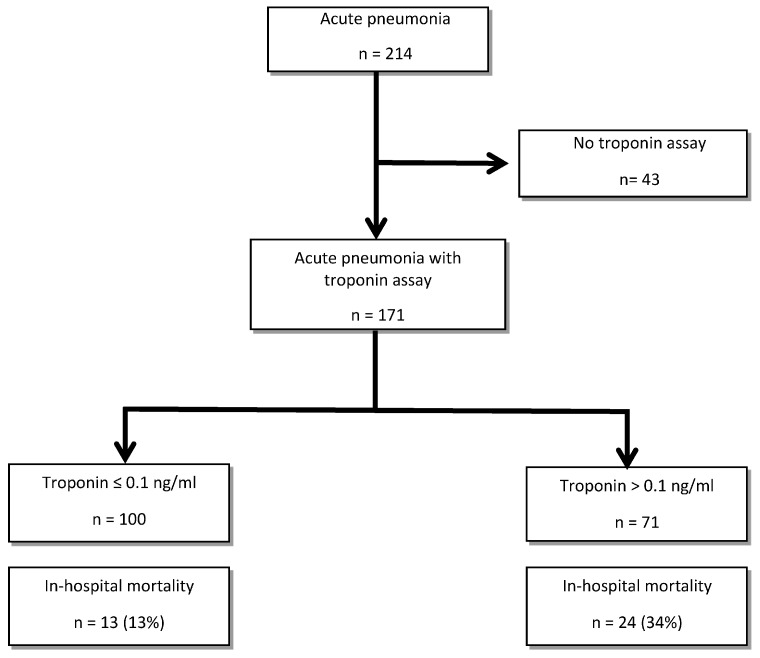
Flow chart.

Inclusion was limited to patients for whom a cardiac troponin I dosage (Dimension Vista luminescent oxygen channeling (LOCI^TM^) troponin I assay, Siemens [23]) was performed in the 72 h following AP diagnosis (Figure 1). Given the high frequency and the atypical clinical presentation of post-infectious MI in this older population [13], local recommendations suggest a screening for myocardial infarction at admission of older patients with AP, by a systematic electrocardiogram and troponin Ic sampling. Patients with a diagnosis of myocardial infarction during the hospitalization prior to AP diagnosis were not included.

This observational study was conducted in accordance with the Declaration of Helsinki and National standards. The Ethics Committee of our institution approved this study.

### 2.2. Definitions

AP was considered community-acquired pneumonia (CAP) or healthcare-associated pneumonia (NHAP) if the first clinical signs appeared at home or at a nursing home, respectively. Pneumonia was considered late-onset hospital-acquired pneumonia (HAP) if the first clinical signs appeared at least 5 days after admission [24].

All events occurring after AP were extracted from the medical records and adjudicated by the study coordinator according to current guidelines and pre-specified criteria.

Myocardial infarction and myocardial injury were defined by the 4th universal definition of myocardial infarction [16]. A cardiac troponin value above the 99th percentile of the upper reference limit (i.e., cardiac troponin I > 100 ng/L) determined myocardial injury. Myocardial infarction diagnosis was retained only in the presence of new ischemic ECG changes, clinical symptoms, or imaging evidence of myocardial ischemia. Without such signs or symptoms, troponin elevation was considered non-ischemic myocardial injury.

CV mortality was defined as a fatal myocardial infarction, fatal stroke, fatal pulmonary embolism, death due to cardiogenic shock or ventricular rhythm disorders, or sudden unexpected death.

Sepsis and septic shock were defined according to the criteria of the Third International Consensus for Sepsis and Septic Shock [25]. We used quick SOFA score to diagnose sepsis (2 out of 3 of the following criteria: respiratory rate >= 22/min, altered mental status, and systolic blood pressure <= 100 mm Hg) and septic shock was defined as the association of 3 criteria (hypotension, elevated lactate level, and a sustained need for vasopressor therapy). Acute respiratory distress syndrome (ARDS) was defined using the Berlin definition [26]. Bleeding was defined using criteria from the Bleeding Academic Research Consortium (BARC) [27].

### 2.3. Recorded Data

For each subject, we recorded demographic, clinical, and laboratory data including age, gender, place of residence, World Health Organization performance status score [28], CV risk factors, underlying diseases including CV history (coronary artery disease, stroke, congestive heart failure, atrial fibrillation), Charlson comorbidity index (CCI) [29], Pneumonia Severity Index (PSI) [30], CURB-65 score [31], clinical presentation at admission, in-hospital outcomes including septic, respiratory, CV and other complications, hospital death, and death at one year. Follow-up at one year was systematically obtained through a phone call to the patient, or if unsuccessful, to their relatives. Only 4 patients (2%) were lost to follow-up at one year and were excluded from the analysis. Uremia, creatininemia, albuminemia, C-reactive protein, procalcitonin, plasma N-terminal pro brain natriuretic peptide (NT-proBNP), hemoglobin levels, and white blood cell counts were taken at admission or, failing that, within 72 h. Microbiological documentation obtained by blood cultures or respiratory samples was also recorded, as described elsewhere [32].

### 2.4. Data Analysis

We compared patients with myocardial injury (i.e., at least one troponin I dosage > 100 ng/L in the 72 h following AP diagnosis) to patients with troponin I ≤ 100 ng/L. Continuous variables were expressed as medians and interquartile ranges, and categorical variables as numbers and percentages. Continuous variables were compared using the Mann–Whitney U test, and categorical variables were compared using the Chi-square test and Fisher test where appropriate. Factors associated with in-hospital and one-year mortality were evaluated in multivariate analysis. Logistic regression analysis was performed to assess the association of troponin elevation on mortality after adjustment on pre-specified prognostic factors (age, CCI, WHO performance status, PSI, CURB-65), according to the literature [29,30,31]. To compare the accuracy of markers to predict death, we constructed receiver operating characteristics (ROCs) and determined the area under the curve (AUC). Patients with missing data were excluded from the analyses. Statistical analyses were performed using SPSS 21.0 software (IBM Corp, Armonk, NY, USA). All statistical tests were 2-tailed. Statistical significance was defined as *p* < 0.05.

## 3. Results

### 3.1. Characteristics at Admission

Among the 214 patients with a diagnosis of AP during the study period, we included the 171 (80%) patients with an available troponin I dosage. Of the 171 inpatients (median age: 86) included, 71 (42%) presented myocardial injury in the 72 h following AP. Patient characteristics at admission are presented in Table 1. These patients were similar to those without myocardial injury in terms of age and site of AP acquisition. They had similar risk factors, performance status and comorbidities, including frequent underlying CV disease in both groups (coronary artery disease: 34% in the myocardial injury group vs. 23% in the other group, *p* = 0.12), but the myocardial injury group had a more severe clinical presentation, as highlighted by AP prognostic scores (median (interquartile range) Pneumonia Severity Index: 60 (40–95) vs. 45 (30–70), *p* = 0.003; CURB-65 > 2: 68 vs. 51%, *p* = 0.03). Clinical presentation in the myocardial injury group included higher heart and respiratory rates, but temperature did not significantly differ between the two groups.

Biological data at admission are presented in Table 2. Inflammatory parameters tended to be higher in patients with myocardial injury (neutrophils 8.89 (6.41–12.95) vs. 7.45 (4.67–11.59) 10^3^/mm^3^, *p* = 0.04; procalcitonin 1.35 (0.34–5.58) vs. 0.52 (0.13–1.19) ng/L, *p* = 0.003), except for C reactive protein, which was similar in both groups. Lymphocytes were significantly lower in patients with myocardial injury (0.78 (0.49–1.18) vs. 1.04 (0.64–1.49), *p* = 0.01). Nutritional status (albumin 25 (22–29) vs. 27 (24–30) g/L, *p* = 0.04), as well as renal function (creatinine rate: 127 (96–178) vs. 93 (73–121) µmol/L, *p* < 0.001) were significantly more altered in patients with myocardial injury. As expected, NT-proBNP levels were significantly higher in patients with myocardial injury (8066 (4234–16,137) vs. 2117 (899–5379) pg/mL).

AP microbiology was documented in only a minority of patients (Table 2). Gram-negative bacteria, *Staphylococcus aureus* and *Streptococcus pneumoniae* were the most frequent pathogens, without a significant difference in frequency between the two groups.

### 3.2. Outcomes

Troponin elevation was strongly associated with worse in-hospital prognosis, including all-cause mortality (33.8 vs. 13%, *p* = 0.002) (Table 3). CV events such as myocardial infarction (25% vs. 0%, *p* < 0.001), cardiogenic shock (14 vs. 3%, *p* = 0.007) and CV mortality (11.3 vs. 1%, *p* = 0.003) were significantly more frequent during hospitalization in patients with troponin elevation. However, troponin elevation did not predict hospital incidence of acute heart failure after AP (65 vs. 55%, *p* = 0.2). Deaths from respiratory causes and sepsis-related deaths were more frequent in patients with troponin elevation, as was admission to intensive care (30 vs. 13%, *p* = 0.01).

### 3.3. Prognostic Factors

In multivariate analysis (Table 4), after adjustment on other prognostic factors, troponin elevation remained a strong predictive factor of in-hospital mortality (odds ratio (95% confidence interval): 3.32 (1.42–7.73), *p* = 0.005), but not after one year (1.61 (0.77–3.35), *p* = 0.2). Unlike age, CCI and performance status, PSI was independently associated with both in-hospital and one-year mortality after AP.

Table 5 presents the prognostic performance of troponin Ic compared with other biomarkers, the PSI, CURB-65, CCI and other prognostic factors, for the prediction of in-hospital and one-year mortality. Troponin Ic (AUC = 0.64) had a modest prognostic value in the short term (similar to PSI), but it poorly predicted one-year mortality (AUC = 0.57). Conversely, NT-proBNP had only long-term predictive value (AUC = 0.66). Among the biomarkers, albumin predicted both short-term (AUC = 0.72) and long-term (AUC = 0.62) mortality. PSI maintained a modest predictive value in both the short (AUC = 0.67) and long-term (AUC = 0.65).

## 4. Discussion

In this unselected population of older comorbid inpatients with AP, cardiac troponin elevation was frequent and strongly associated with short-term mortality. To our knowledge, this study is the first to investigate the prognostic value of troponin elevation in hospitalized AP patients aged 75 and older. The prevalence of myocardial injury, defined by an elevated troponin level, was evaluated to be 42% in our series, which is similar to previous reports in younger patients with CAP [11,18]. Vestjens et al., in a hospital population of CAP (mean age 56 years), reported elevated cardiac troponin T in 45% of patients, while Cangemi et al., in a somewhat older population (mean age 70 years), found an even higher prevalence of myocardial injury (52%). These two studies, like ours, found that troponin elevation was strongly correlated with PSI. These results are consistent with other studies as well, highlighting that the risk of myocardial infarction after AP is closely linked to PSI [33].

Few studies have assessed whether troponin elevation at AP diagnosis could predict CV events. In older populations at a higher risk of CV events [34], there is a need for an easy-to-use predictive tool for the prevention of CV events. Indeed, there is growing evidence that preventative CV therapies could improve outcomes after AP [35,36,37,38,39]. However, in this frail comorbid population, an individualized assessment of post-AP CV risk is needed to limit the iatrogenic effects of drugs used for CV prevention, including bleeding complications.

In patients presenting an elevated troponin rate at AP diagnosis, we observed a high incidence of in-hospital CV complications, including myocardial infarction in a third of patients. Interestingly, none of the 100 patients with negative troponin at admission had a diagnosis of myocardial infarction during hospitalization. These results suggest that cardiac troponin assay has an excellent negative predictive value for post-AP myocardial infarction. To our knowledge, only one recent study has evaluated the predictive value of cardiac troponin for subsequent CV events after AP [40]. Menendez et al. reported that cardiac troponin was the most predictive biomarker for 30-day incidence of CV events: elevated cardiac troponin T was associated with a nearly three-fold risk of early CV events [40].

Cardiac troponin rates have been associated with short-term mortality in several series, while its long-term prognostic value is more debatable [41]. Other CV biomarkers, including NT-proBNP, could have superior prognostic value in the long term [19], especially when coupled with other biomarkers [42] or prognostic indexes [43].

While other reports found that *Streptococcus pneumonia* was associated with a higher risk of CV complications after AP [8], we found no association between myocardial injury and any specific pathogen. This could be explained in part by a low rate of microbiological documentation and the relatively small sample size of this study.

This study has some limitations. Firstly, 20% of patients were not tested for troponin in the 72 h following AP diagnosis and were thus excluded. The incidence of myocardial injury may therefore have been overestimated. However, previous series found similar results [11,18]. Moreover, this limitation led to the selection of a population at higher CV risk and for whom a troponin dosage was considered necessary by the treating physician. Secondly, our series was based on conventional troponin I dosage, leading to a possible underestimation of myocardial injury compared with other studies using high-sensitivity cardiac troponin T [11,18,44]. The most recent guidelines do indeed recommend the preferential use of high-sensitivity methods [16,45,46]. However, the use of high-sensitivity methods leads to a reduced specificity of troponin for acute myocardial infarction, especially in older patients and in critically ill patients with sepsis [47]. Moreover, recent studies reporting myocardial injury after AP using such methods found similar incidence rates, albeit in younger populations with a lower CV risk [11,18]. Thirdly, the timing of troponin sampling at inclusion was not standardized, and there were potential differences between samples taken at admission or within 72 h. Moreover, this study was performed over a relatively short period (6 months). Additionally, because a second troponin sample was not systematic, acute and chronic myocardial injury were not dissociated in this study, although CV outcomes differ between these two entities [48]. Finally, we cannot exclude that in some cases AP may have been a complication rather than a cause of myocardial injury. Indeed, in congestive heart failure, pulmonary alveolar flooding may lead to a doubled risk of AP, as highlighted in observational studies [49]. However, this pathophysiological consideration does not affect the prognostic value of troponin in AP.

In this older hospitalized population with AP, myocardial injury was identified in nearly half of patients. Troponin elevation at AP diagnosis was a strong predictor of in-hospital CV events and was independently associated with in-hospital mortality. If these results are confirmed, troponin sampling at AP diagnosis could help physicians to perform individualized CV risk evaluations and focus CV prevention strategies in this frail comorbid population. Further research is needed to evaluate the benefit of such strategies.

## Figures and Tables

**Table 1:** Characteristics at admission of older in-patients with acute pneumonia (*n* (%) or median (interquartile range)).

	Troponin ≤ 100 ng/L *n* = 100	Troponin > 100 ng/L *n* = 71	*p*
Age (years)	86 (81–90)	85 (91–90)	0.7
Men	36 (36)	36 (51)	0.05
Community-acquired pneumonia	59 (59)	43 (61)	0.6
Nursing home-acquired pneumonia	31 (31)	18 (25)	
Hospital-acquired pneumonia	10 (10)	10 (14)	
Charlson Comorbidity index	2 (2–4)	3 (2–5)	0.3
Performance status > 2	68 (68)	45 (64)	0.8
Pneumonia Severity Index	45 (30–70)	60 (40–95)	0.003
CURB-65 > 2	51 (51)	48 (68)	0.03
**Cardiovascular risk factors**			
Actively smoking	9 (9)	6 (8)	0.9
High blood pressure	77 (77)	52 (73)	0.6
Dyslipidemia	23 (23)	19 (27)	0.6
Diabetes	24 (24)	18 (25)	0.8
**Underlying disease**			
Stroke	13 (13)	10 (14)	0.8
Coronary artery disease	23 (23)	24 (34)	0.1
Atrial fibrillation/flutter	36 (36)	27 (38)	0.8
Congestive heart failure	16 (16)	6 (9)	0.2
Chronic respiratory disease	32 (32)	12 (16)	0.03
Chronic kidney disease	12 (12)	7 (10)	0.7
Thromboembolism	8 (8)	8 (11)	0.5
Cognitive disorders	30 (30)	18 (25)	0.5
Hematologic malignancy	15 (15)	6 (8)	0.2
Neoplasia	18 (18)	16 (22)	0.5
**Clinical presentation**			
Heart rate (bpm)	86.5 (71–102)	98.5 (78–110)	0.02
SBP (mmHg)	133 (110–165)	135 (104–161)	0.6
DBP (mmHg)	66.5 (57–81)	63 (58–82)	0.7
Temperature (°C)	37.4 (36.5–38.3)	37.8 (36.9–38.7)	0.2
O_2_ saturation (%)	92 (88–95)	91 (80–95)	0.3
Confusion	47 (47)	31 (44)	0.4
Dyspnea	71 (71)	52 (74)	0.6
Respiratory rate > 30/min	26 (26)	35 (49)	0.003

DBP: Diastolic blood pressure, SBP: systolic blood pressure.

**Table 2:** Biological and microbiological data at admission of older in-patients with acute pneumonia (*n* (%) or median (interquartile range)).

	Troponin ≤ 100 ng/L *n* = 100	Troponin > 100 ng/L *n* = 71	*p*
**Biology**			
White blood cells (10^3^/mm^3^)	9.75 (6.57–19.95)	10.75 (7.77–15.15)	0.1
Neutrophils (10^3^/mm^3^)	7.45 (4.67–11.59)	8.89 (6.41–12.95)	0.04
Monocytes (10^3^/mm^3^)	0.81 (0.57–1.14)	0.7 (0.53–1.09)	0.2
Lymphocytes (10^3^/mm^3^)	1.04 (0.64–1.49)	0.78 (0.49–1.18)	0.01
Haemoglobin (g/dL)	12.2 (10.8–13.4)	12.3 (10.9–13.4)	0.8
Troponin I (ng/L)	0 (0–100)	500 (200–2000)	<0.001
NT-pro Brain Natriuretic Peptide (pg/mL)	2117 (899–5379)	8066 (4234–16,137)	<0.001
Urea (mmol/L)	8.8 (6.12–13.3)	10.9 (7.9–19)	0.008
Creatinine (µmol/L)	93 (73.5–121.2)	127 (96–178)	<0.001
Albumin (g/L)	27 (24–30)	25 (22–29)	0.04
C-reactive protein (mg/L)	172 (118–219)	174 (135–240)	0.6
Procalcitonin (ng/L)	0.52 (0.13–1.19)	1.35 (0.34–5.58)	0.003
**Microbiology**			
*Streptococcus pneumoniae*	9 (9)	5 (7)	0.6
*Staphylococcus aureus*	5 (5)	6 (8)	0.4
Gram-negative bacteria	23 (23)	13 (18)	0.5
Other bacteria	2 (2)	1 (1)	1
Influenza virus	1 (1)	1 (1)	1
Other viruses	3 (3)	3 (4)	0.7

**Table 3:** In-hospital outcomes after acute pneumonia in older inpatients (*n* (%) or median (interquartile range)).

	Troponin ≤ 100 ng/L *n* = 100	Troponin > 100 ng/L *n* = 71	*p*
**Sepsis**			
Sepsis	16 (16)	13 (18)	0.7
Septic shock	8 (8)	16 (22)	0.007
Sepsis-related death	3 (3)	11 (15)	0.003
**Respiratory events**			
Pleural effusion	20 (20)	16 (22)	0.7
ARDS	5 (5)	8 (11)	0.1
Non-invasive ventilation	12 (12)	21 (30)	0.004
Endotracheal intubation	8 (8)	15 (22)	0.01
Respiratory death	8 (8)	13 (18)	0.04
**Cardiovascular events**			
Acute heart failure	55 (55)	46 (65)	0.2
Cardiogenic shock	3 (3)	10 (14)	0.007
Myocardial infarction	0	25 (35)	<0.001
New atrial fibrillation	11 (11)	4 (6)	0.3
Cardiac arrest	0	6 (8)	0.003
Stroke	7 (7)	2 (3)	0.3
Pulmonary embolism	6 (6)	2 (3)	0.3
Deep vein thrombosis	6 (6)	5 (7)	0.8
Acute peripheral ischemia	0 (0)	2 (3)	0.2
Cardiovascular death	1 (1)	8 (11)	0.003
**Other events**			
Acute kidney failure	40 (40)	42 (66)	0.001
Anaemia	38 (38)	28 (39)	0.8
Bleeding *	9 (9)	14 (20)	0.04
Blood transfusion	13 (13)	11 (15)	0.6
Length of hospital stay (days)	14 (9–26)	16 (8–24)	0.9
Intensive care requirement	13 (13)	21 (30)	0.01
Palliative care requirement	11(11)	14 (20)	0.1
**Hospital death**	13 (13)	24 (34)	0.002

ARDS: acute respiratory distress syndrome; * Bleeding Academic Research Consortium (BARC) definition > 1.

**Table 4:** Multivariate analysis of factors associated with in-hospital and one-year mortality after acute pneumonia in older inpatients (*n* (%) or median (interquartile range)).

	In-Hospital Mortality *n* = 37		One-Year Mortality *n* = 85	
	Odds Ratio (95% CI)	*p*	Odds Ratio (95% CI)	*p*
Charlson Comorbidity index	1.02 (0.83–1.25)	0.9	1.03 (0.86–1.24)	0.7
Age (year)	0.98 (0.91–1.05)	0.6	1.05 (0.99–1.11)	0.1
Troponin Ic (ng/L)	3.32 (1.42–7.73)	0.005	1.61 (0.77–3.35)	0.2
Performance Status	1.47 (0.85–2.52)	0.2	2.45 (1.50–4.02)	<0.001
Pneumonia Severity Index	1.02 (1.01–1.04)	0.003	1.03 (1.01–1.05)	<0.001

CI: Confidence Interval.

**Table 5:** Area Under the Receiver Operating Characteristic Curve (AUC) for in-hospital mortality and one-year mortality comparing the main prognostic factors after acute pneumonia.

	In-Hospital Mortality		One-Year Mortality	
	AUC (95% CI)	*p*	AUROC (95% CI)	*p*
**Biomarkers**				
Troponin Ic	0.64 (0.54–0.74)	0.008	0.57 (0.49–0.66)	0.09
NT-pro Brain Natriuretic Peptide	0.59 (0.47–0.70)	0.2	0.66 (0.57–0.75)	0.001
Urea	0.66 (0.56–0.76)	0.002	0.63 (0.54–0.71)	0.003
1/Albumin	0.72 (0.62–0.82)	<0.001	0.62 (0.52–0.71)	0.02
C reactive protein	0.43 (0.31–0.54)	0.2	0.49 (0.40–0.58)	0.9
**Main prognostic factors**				
Pneumonia Severity Index	0.67 (0.58–0.77)	0.001	0.65 (0.57–0.74)	0.001
CURB-65	0.59 (0.48–0.69)	0.1	0.56 (0.48–0.65)	0.1
Charlson Comorbidity index	0.55 (0.44–0.65)	0.4	0.54 (0.45–0.62)	0.4
Age	0.46 (0.37–0.57)	0.5	0.60 (0.51–0.68)	0.02
Performance Status	0.55 (0.44–0.67)	0.3	0.67 (0.58–0.75)	<0.001

CI: Confidence Interval.
